# Subtracted Diversity Array Identifies Novel Molecular Markers Including Retrotransposons for Fingerprinting *Echinacea* Species

**DOI:** 10.1371/journal.pone.0070347

**Published:** 2013-08-05

**Authors:** Alexandra Olarte, Nitin Mantri, Gregory Nugent, Edwin C. K. Pang

**Affiliations:** School of Applied Sciences, Health Innovations Research Institute, RMIT University, Melbourne, Victoria, Australia; Queensland Institute of Medical Research, Australia

## Abstract

*Echinacea,* native to the Canadian prairies and the prairie states of the United States, has a long tradition as a folk medicine for the Native Americans. Currently, *Echinacea* are among the top 10 selling herbal medicines in the U.S. and Europe, due to increasing popularity for the treatment of common cold and ability to stimulate the immune system. However, the genetic relationship within the species of this genus is unclear, making the authentication of the species used for the medicinal industry more difficult. We report the construction of a novel Subtracted Diversity Array (SDA) for *Echinacea* species and demonstrate the potential of this array for isolating highly polymorphic sequences. In order to selectively isolate *Echinacea*-specific sequences, a Suppression Subtractive Hybridization (SSH) was performed between a pool of twenty-four *Echinacea* genotypes and a pool of other angiosperms and non-angiosperms. A total of 283 subtracted genomic DNA (gDNA) fragments were amplified and arrayed. Twenty-seven *Echinacea* genotypes including four that were not used in the array construction could be successfully discriminated. Interestingly, unknown samples of *E. paradoxa* and *E. purpurea* could be unambiguously identified from the cluster analysis. Furthermore, this *Echinacea*-specific SDA was also able to isolate highly polymorphic retrotransposon sequences. Five out of the eleven most discriminatory features matched to known retrotransposons. This is the first time retrotransposon sequences have been used to fingerprint *Echinacea*, highlighting the potential of retrotransposons as based molecular markers useful for fingerprinting and studying diversity patterns in *Echinacea*.

## Introduction

*Echinacea* (family: Asteraceae) is a North American genus that is widely recognized for its medicinal uses. The species of this genus, particularly *E. angustifolia* DC., have been traditionally used by the Plain Indians for relieving toothache, coughs, colds, sore throats, snakebites and as a painkiller [1]. Contemporarily, *Echinacea* has been recognized for its ability to stimulate the immune system and to effectively moderate the incidence, duration and severity of symptoms associated with common cold [2]–[4].

*E. angustifolia*, *E. purpurea* (L.) Moench. and *E. pallida* (Nutt.) Nutt., are the three main species commonly used for extracts or whole-plant products in the herbal medicine industry. In Australia where is reported that 50% of the population uses complementary and alternative medicine; the annual consumption of *E. purpurea* in 2000 was 80MT, 15 to 20MT of *E. angustifolia* and 1MT of *E. pallida* [4]. More recently, increases in the 2009 sales on *Echinacea* supplements (11.26% in the natural and health food channels) were seen in the United States as a possible consequence of the global concern for the Influenza A (H1N1) virus [5]. This increase in the demand seen over the last decades has created several problems for trade regulation, especially enforcing the sale of authentic species. Identification of correct species is still problematic due to morphological similarities between species and to the introduction of wild collected seeds into cultivation without proper authentication [6]. In Europe for example, cultivated *E. pallida* was sold as *E. angustifolia* as a result of the high morphological variability found within populations which made difficult the use of the identification keys proposed by McGregor’s taxonomic classification [7].

McGregor’s taxonomical classification, established in 1968, recognized nine species and four varieties of *Echinacea* [8]. However, this classification has inconsistencies among the descriptions that present practical difficulties when using his keys [7]. A revision of this classification [7] recognized four species and eight varieties based on morphometric analyses. Although both classifications are based on relatively minor differences among characters; McGregor’s classification continues to be widely used by botanists and herbalists until other studies are capable of providing greater support for re-classification. Similarly, for commercial purposes, the classification proposed by McGregor is still being employed since any re-labeling of the *Echinacea* products will bring cost to the industry and will create a confusion among the customers [9]. For this study, we also used McGregor’s classification as a reference.

Molecular fingerprinting has been employed to find independent support for the morphology-based classifications; however these results are also contradictory. For example, Amplified Fragment Length Polymorphism (AFLP) markers have been employed to fingerprint all species in this genus [10], [11]. The first study [10] found two major clades, one containing *E. purpurea, E. sanguinea* Nutt. and *E. simulata* McGregor. and the other containing the remaining species. Their data indicated that all *Echinacea* taxa are closely related as shown by McGregor. In contrast, the second AFLP study [11] found support for classification of *Echinacea* into four species as proposed by Binns’s classification but could not support their classification in eight varieties. The discrepancy between these two studies can probably be attributed to the primer combination used and the number of individuals sampled.

Phylogenetic studies have also been performed for this genus; however they were unable to resolve the species-level relationships due to the low levels of molecular divergence found in the selected loci. For instance, the sequence divergence of two chloroplast (*trn*S and *trn*G) and three nuclear loci [*Adh* (alcohol dehydrogenase), *Ces*A (cellulose synthase) and *GPAT* (3-phosphate acetyltransferase)] were unable to provide a resolved topology or congruent hypotheses about species-level relationships [12]. In addition, sequence divergence of the internal transcribed spacer (ITS) and intervening 5.8S regions was found low (0.18% to 3.2%) within *Echinacea* species. Interestingly, several species had identical ITS-2 sequences [13]. Therefore, different molecular studies have also been unable to completely resolve the relationships among *Echinacea* species.

In order to clarify the genetic relationships within this genus, there is a need for molecular techniques that are not only able to clearly distinguish species and varieties but which are also able to overcome the main limitations of polymerase chain reaction (PCR)-based techniques, i.e., the assumption that co-migrating fragments are homologous. Previous AFLP studies have indicated that this assumption is not always valid since it was found that co-migrating polymorphic bands from different species and varieties of *Echinacea* were not homologous [14]. This is a significant disadvantage and therefore the data obtained from these techniques are usually inappropriate for phylogenetic studies.

A new technique called Subtracted Diversity Array (SDA) combines an alternative Suppression Subtractive Hybridization (SSH) approach with high-density microarray to increase the chances of finding polymorphic features [15], [16]. The alternative SSH involves the pooling of gDNA representations and single subtraction instead of making multiple pair-wise subtractions between the species as proposed by other subtraction techniques [17]. Multiple pair-wise subtractions could be costly and time consuming; for example, a total of four subtractions had to be performed in order to fingerprint only six *Dendrobium* species [18]. Furthermore, SDA could potentially be a superior technique for assessing the genetic relationships among *Echinacea* species since it does not require previous DNA sequence information and has shown to be capable of differentiating closely-related species of the same genus [19], [20]. For instance, the first genera-specific SDA, constructed by a stringent subtraction between a pool of *Salvia* species and a pool of angiosperms and non-angiosperms, was able to fingerprint 15 *Salvia* genotypes and to construct a hierarchical cluster that was consistent with the geographical origin of the species [20]. Thus, this technique has the ability to selectively isolate polymorphic *Echinacea*-specific sequences.

Furthermore, the molecular profile obtained with the SDA could be employed for the identification of potential molecular markers that could be genotype-specific or that could be associated with the bioactive compound content. There is a recent study [21], in which metabolite profiles for 40 lines of *Echinacea* were generated by high performance liquid chromatography (HPLC). The lines analyzed represented a broad geographical and morphological background and were also used in the phylogenetic study described above [12]. It will be of interest to use these same lines to develop the molecular profiles with the aim of identifying potential molecular markers associated with the production of bioactive compounds and to compare if there is any resemblance among the dendrograms obtained with molecular and chemical analyses. This study reports the construction of an *Echinacea-*specific SDA and demonstrates its ability to assess the genetic relationships among *Echinacea* genotypes. Additionally, we identified potential nuclear molecular markers five of which were recognized as retrotransposons.

## Materials and Methods

### Plant Material

In order to develop a gDNA representation for the subtraction, DNA from a total of 143 species including angiosperms and non-angiosperms were soured **(Table S1)**. Non-angiosperms were collected from Toolangi State Park, Victoria (Australia) and identified [22]. The permit to collect protected flora was granted by Department of Sustainability and Environment. Angiosperms were obtained only from verified nursery species; a total of 118 species were sourced to represent all angiosperm clades.

Additionally, a total of 24 lines were used to represent the *Echinacea* genus. Five *Echinacea* species, as mentioned by McGregor, (*E. angustifolia*, *E. paradoxa* (Norton) Britton, *E. pallida*, *E. purpurea* and *E. tennesseensis* (Beadle) Small.) were obtained from three different sources. The other four species (*E. atrorubens* Nutt., *E. laevigata* (Boynton & Beadle) Blake, *E. sanguinea*, *E. simulata*) could not be obtained as quarantine restrictions prevented importation into Australia**.** Nineteen of the 24 lines were selected from the germplasm collection of the U.S. National Plant Germplasm System maintained by the USDA-ARS North Central Regional Plant Introduction Station (NCRPIS) **(** **Table 1** **)**. These 19 lines were previously used in two independent studies [12], [21]. The other remaining lines were obtained from Botanical Resources Australia (Tasmania) and from verified specimens from a specialized plant nursery (The Diggers Club, Dromana Victoria) **(** **Table 1** **)**.

**Table 1:** Description of the *Echinacea* species used for DNA extraction and development of genome representations.

Taxon (McGregor, 1968)^a^	Accession numberb,c,d	Abbreviation^e^
***E. angustifolia*** ** DC.**	PI631267 (OK)	ang 267
***E. angustifolia*** ** DC. var. ** ***angustifolia***	PI631272 (OK)	ang-ang 272
	PI631285 (IA)	ang-ang 285
	PI631318 (KS)	ang-ang 318
***E. angustifolia*** ** DC. var. ** ***strigosa*** ** McGregor**	PI631266 (OK)	ang-str 266
	PI631320 (OK)	ang-str 320
***E. pallida*** ** (Nutt.) Nutt.**	PI631275 (OK)	pal 275
	PI631290 (IA)	pal 290
	PI631293 (AR)	pal 293
	PI631296 (MO)	pal 296
	PI631315 (NC)	pal 315
	“Hula dancer”^c^	N/A
***E. paradoxa*** ** (Norton) Britton var. ** ***neglecta*** ** McGregor**	PI631263 (OK)	px-neg 263
	PI631264 (OK)	px-neg 264
	PI631265 (OK)	px-neg 265
***E. paradoxa*** ** (Norton) Britton var. ** ***paradoxa***	PI631301 (MO)	px-px 301
	PI631321 (MO)	px-px 321
***E. purpurea*** ** (L.) Moench**	PI631307 (MO)	pur 307
	PI631313 (NC)	pur 313
	PI633669 (LA)	pur 669
	“Double Decker”^c^	N/A
	“White purpurea”^c^	N/A
	“purpurea” c	N/A
***E. tennesseensis***	*E. tennesseensis* d	N/A
**Putative hybrid. ** ***E. paradoxa*** ** var. ** ***paradoxa*** ** and ** ***E. pallida*** a	PI631294 (AR)^a^	hyb 294
***E. angustifolia*** a	Plot 9a,d (OR)	ang plot 9
***E. pallida*** a	Plot 5a,d (Germany)	pal plot 5
***E. purpurea*** a	Plot 10009a,d. (Commercial crop )	pur plot 10009

Notes: AR, Arkansas; IA, Iowa; KS, Kansas; LA, Louisian a; MO, Missouri; NC, North Carolina; OK, Oklahoma; OR, Oregon; SC, South Carolina; TN, Tennessee; VA, Virginia.

a *Echinacea* not included in the SDA development.

b *Echinacea* with PI accessions numbers were obtained from the germplasm collection in the U.S. National Plant Germplasm System.

c *Echinacea* verified specimens obtained from a specialized plant nursery (The Diggers Club. Dromana VIC).

d *Echinacea* obtained from the Botanical Resources Australia (Tasmania).

e The abbreviated names are used to refer to the accessions in the figures.

### DNA Extraction and Development of Tester and Driver Pools

Total DNA was extracted following our previously described method [20] which combines a modification of the standard CTAB (cetylmethylammonium bromide) procedure with an additional clean up performed using the DNeasy® column of the DNeasy® Plant Mini Kit (Qiagen). Subsequently, all DNA samples were pooled based on the Angiosperm Phylogeny Group (2009) classification [23] in order to obtain representations of the following seven groups: all *Echinacea* species (subtraction pool), Asterids (excluding Asteraceae), non-angiosperms, Monocots, Magnoliids, Rosids, and Eudicots not belonging to the Rosids or Asterids (Eudicots and Core Eudicots) **(** **Table 1** **and S1)**. About 10 µg of DNA was bulked for each representation, with each pool having equal amounts of gDNA per species. Subsequently, each pool was separately concentrated using the DNeasy® column of the DNeasy® Plant Mini Kit (Qiagen). The concentration and purity of the DNA pools were evaluated spectrophotometrically whilst the quality/integrity was assessed by 1.5% agarose gel electrophoresis.

### Subtraction, Library and SDA Construction

The method used for subtraction, library and microarray construction was prepared using our previously described method [16].

Subtraction was performed using the PCR-Select™ Bacterial Genome Subtraction Kit (Clontech), following the manufacturer’s protocol. The *Echinacea* pool (tester) was prepared by mixing equal amounts of DNA extracted from the 24 genotypes mentioned above. The driver pool was formed by bulking 700 ng of each non-*Echinacea* representation [Asterids (excluding Asteraceae), non-angiosperms, Monocots, Magnoliids, Rosids, and Eudicots not belonging to the Rosids or Asterids (Eudicots and Core Eudicots)] **(Table S1)**. It is important to note that the two subtraction hybridizations were performed using a tester:driver ratio of 1∶60.

The subtracted product was then purified using the QIAquick PCR Purification Kit (Qiagen). Then, approximately 100 ng of the purified PCR products were ligated into the pGEM®-T Easy vector (Promega) and transformed into heat-shock competent *Escherichia coli* JM109 (Promega) according to the user manual. Positive transformation was determined by PCR amplification of the cloned insert using the nested primers from the subtraction kit. A total of 283 positive recombinant *E. coli* clones were finally diluted in one volume of sterile glycerol and stored at −70°C.

The 283 *Echinacea*- specific DNA clones were amplified in 100 µl PCR reactions using nested primers 1 and 2R (Clontech). The template used for the amplification was obtained by mixing 10 µl of bacterial cell culture with 10 µl of MilliQ water and then heated at 100°C for 10 min to disrupt the cells and release the plasmid DNA. Then 1.5 µl of this sample was used as template. After amplification, PCR products were precipitated in 96% ethanol and 3 M sodium acetate (pH 5.2). The precipitation was carried out at −20°C overnight. The pellets obtained were washed with 70% ethanol, air dried and resuspended in 10 µl of 50% DMSO.

The 283 clones together with the controls were gridded on Corning® GAPS II coated slides (Corning Incorporated Life Sciences, Acton, MA) using a BioRobotics® MicroGrid II Compact arrayer (Genomic Solutions) at RMIT University, Australia. The positive controls included three housekeeping genes (ribulose-1,5-bisphosphate carboxylase/oxygenase, ribosomal RNA and chlorophyll a/b binding protein) sourced from *Cicer arietinum* [24]. A single printed slide was used to perform two hybridization experiments, where each hybridization reaction was tested with 5 sub-arrays (each sub-array composed of the 283 clones and 17 controls).

### Target Synthesis and SDA Hybridization

The SDA was firstly validated by performing separate hybridizations with the biotin-labeled DNA from the *Echinacea* (tester) and non-*Echinacea* (driver) pools. Subsequently, fingerprints of each *Echinacea* genotype were obtained by hybridizing their biotin-labeled DNA to the array. Labeling of the targets and hybridization of the microarray slides were mainly performed as described in our previous method [20]. However, slight modifications were performed. Hybridization of the biotin-labeled DNA of each *Echinacea* accession was performed at 47°C, instead of the 42°C, to facilitate a higher level of discrimination between the lines. Further, all hybridizations were performed with five technical replicates (sub-arrays) and two biological replicates, for a total of ten data points per array feature.

After hybridization, the coverslips were removed and the slides were washed once in 1 x SSC and 0.1% SDS at 37°C for 8 min, once in 1×SSC and 0.1% SDS at 40°C for 5 min, once in 0.1×SSC and 0.1% SDS at 35°C for 5 min and once in 0.1×SSC at 35°C for 5 min. Subsequently, detection of the biotinylated DNA targets bound on the array was performed by a protocol modified from Mirus Label IT® µArray® Biotin Labeling Kit [25]. Briefly, the slides were washed once in 6×SSPE-T buffer (0.9 M NaCl, 0.06 M NaH_2_PO_4_·H_2_O, 0.006 M EDTA, 0.005% Triton X-100, pH 7.4) at room temperature for 5 min. Subsequently, the detection solution (0.8 µl of 25 µg/µl of BSA, 0.5 µl of 0.8 µg/µl streptavidin-labeled Cy^TM^3 dye (Amersham Pharmacia, UK), made to 200 µl with 6×SSPE-T) was applied to the wet surface of the slide and covered by a 25×60 mm lifter coverslip (Grale Scientific, Australia) to evenly distribute the solution. The slides were incubated at 37°C for 40 min in a waterproof hybridization chamber in the dark. Finally, the slides were washed three times in 6×SSPE-T buffer for 5 min, rinsed with deionized water and dried with an air gun.

A total of 27 genotypes were fingerprinted; 23 of which were used to construct the subtraction pool (excluding *E. purpurea* “Double Decker”) and four additional genotypes that were not used for the SDA construction **(** **Table 1** **)**.

### Scanning and Data Analysis

The scanning and data analysis were performed as described [20]. Slides were scanned with a ScanArray Gx (PerkinElmer Life and Analytical Sciences, Downers Grove, IL) microarray scanner in conjunction with the supplied software. The slides were scanned with a resolution of 10 µm at 532 nm (Cy3, green laser) and at 50% photomultiplicator (PMT) gain whilst keeping background noise low. The scanned array was quantified using PerkinElmer ScanArray Express software v 4.0. The program individually quantified the signal intensity of each spot using adaptive circle method and normalized the data using the LOWESS function. Local background was subtracted during quantitation. The signal-to-noise ratio obtained for each spot was considered to have the most accurate background correction since it also accounted for variations in background intensity over the array. Quantified data was exported to Microsoft Excel (Microsoft) and abnormal spots that were not automatically flagged by the software were flagged manually.

Data analysis included the calculation of the mean signal-to-noise ratio (mean signal intensity) for each feature between the five technical replicates, normalization across the slides and combination (average) of the biological replicates to produce a single value per feature. This entire data set has been deposited in Gene Expression Omnibus (GSE44683).

The data set was then transferred to PASW Statistics 18 to perform a hierarchical cluster analysis of the 27 *Echinacea* genotypes. The dissimilarity dendrogram was generated using the average distance linkage between-groups and Square Euclidian metrics. Additionally, the normalized mean signal intensity was used for principal component analyses (PCA) and correlate bivariate analysis using MINITAB® Release 14.1 and PASW Statistics 18, respectively. These two analyses together with the magnitude of the variance calculated for each feature across the 27 fingerprints were able to distinguish the most discriminatory features useful for fingerprinting.

Data obtained from a previous study [21] on metabolomic profiling of *Echinacea* genotypes was used for correlation analyses with the hybridization data. The relative abundance of 43 lipophilic metabolites in roots from 6-month-old plants was correlated with the normalized mean signal of the full feature set by performing Pearson bivariate correlations (SPSS version 17.0) and regression analysis (Microsoft Excel). The correlations were performed for only **19 lines** that were shared by the two studies.

### Sequencing of Selected most Discriminatory and Species-specific Features

The cloned inserts were re-amplified from the corresponding isolated plasmids using SP6/T7 primers. Amplification products were bi-directionally sequenced at Macrogen Inc. (Korea). Vector and primer sequences were removed and nucleic acid and protein homology searches were performed using blastN and blastX programs through the National Center of Biotechnology Information (www.ncbi.nlm.nih.gov/BLAST/). All sequences have been deposited in the EMBL Nucleotide Sequence Database (Accession number HF585700 to HF585713).

## Results

### Subtraction Efficiency and Validation of the Microarray

The *Echinacea*-specific microarray was first validated by determining the subtraction efficiency *i.e.*, if the constructed array contained only *Echinacea*-specific sequences. For this, one hybridization with the gDNA pool from 24 *Echinacea* lines (tester pool) and another separate hybridization with the gDNA pool of 142 species representing the non- *Echinacea* angiosperms and non-angiosperms (driver pool) were performed. Eight (3%) positive features were found after hybridizing the driver target with the array, indicating that the subtraction procedure was able to isolate *Echinacea*-specific DNA sequences with 97% efficiency. Based on the above results, it may be implied that the *Echinacea* array had a lower percentage (3%) of sequences homologous to the driver which may represent the non-subtracted sequences.

### Fingerprinting the Twenty-seven *Echinacea* Genotypes

Fingerprints for twenty-seven *Echinacea* lines were obtained by individually hybridizing their gDNA onto the *Echinacea*-specific array. The fingerprints were representative of five species, namely, *E. angustifolia*, *E. paradoxa*, *E. pallida*, *E. purpurea* and *E. tennesseensis* [8]. Out of the twenty-seven, four fingerprints corresponded to lines (Plot 9, 5, 10009 and accession PI631294) which were not used in the construction of the original subtraction pool from which the subtraction technique was performed (**Table 1**).

**Figure 1:**
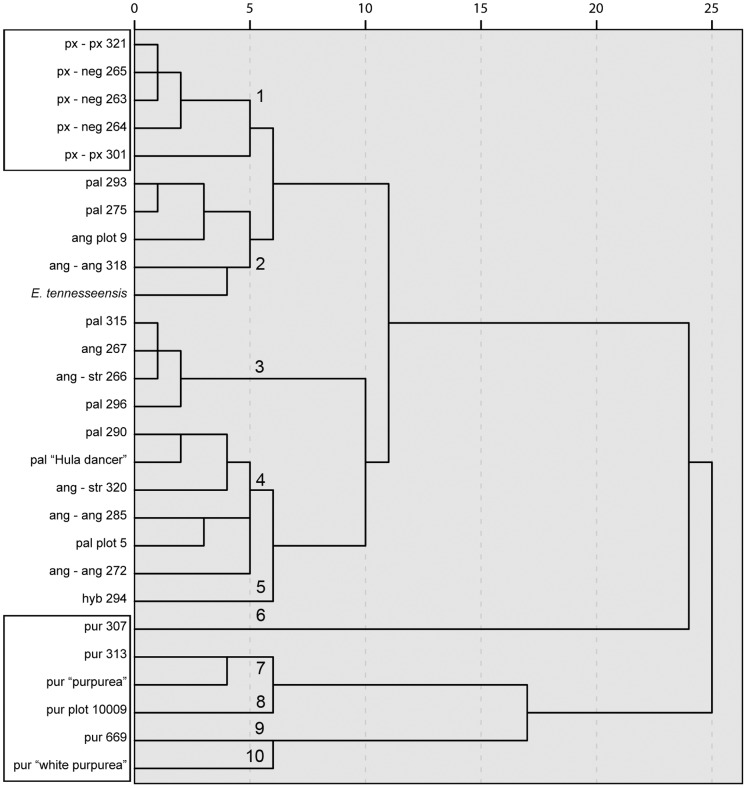
Dissimilarity dendrogram for the SDA hybridization patterns of 27 *Echinacea* genotypes using the 283 features. The steps of the dendrogram (Squared Euclidian distance, between groups linkage) show the combined clusters and the values of the distance coefficients at each step; the values have been rescaled to numbers between 0 and 25, preserving the ratio of the distances between the steps. The equivalents of the abbreviated names used for each of the genotypes are shown in Table 1.

The hierarchical cluster analysis performed using the signal intensities of the 283 features provided a clear differentiation between the twenty-seven *Echinacea* genotypes tested (**Figure 1**). This dissimilarity dendrogram produced ten clusters at the cut off-point of 5. Cluster 1 included all genotypes belonging to *E. paradoxa*. Cluster 2 contained two genotypes of *E. pallida*, two *E. angustifolia* genotypes and *E. tennesseensis.* Cluster 3 and 4 included genotypes from *E. angustifolia* and *E. pallida*. Cluster 5 contained only the putative *E. paradoxa* var. *paradoxa* and *E. pallida* hybrid. Clusters 6 through 10 contained all genotypes belonging to *E. purpurea*.

### Identification of the most Discriminatory and Species-specific Features

**Figure 2:**
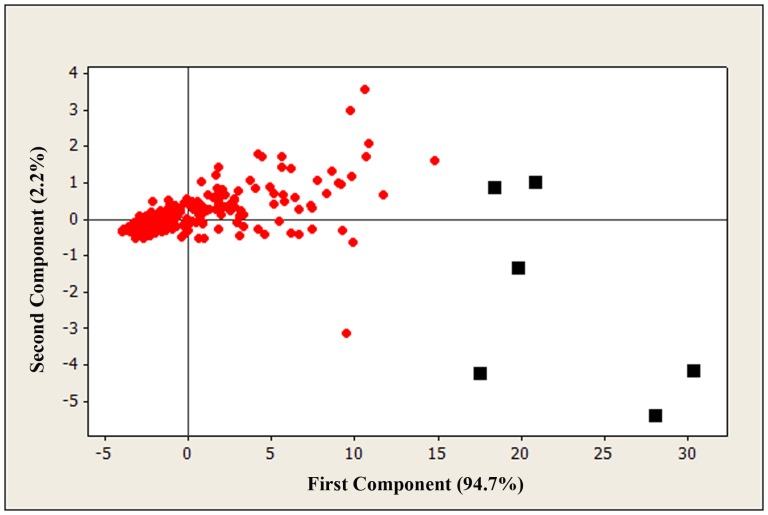
Principal component analysis plot for the 283 features. The first principal component accounts for 94.7% of variation and the second component explained only 2.2% of variation. The squares represent features that account for most of the variability across the genotypes.

Identification of the most discriminatory and species-specific features was performed by a series of statistical analyses using the normalized mean signal intensity of the 283 features across the 27 genotypes. Principal components analysis (PCA) was performed in order to identify the features that accounted for most of the variability found across the genotypes. Principal component analysis indicated that a high percentage of the variation (96.9%) may be explained by the first two components. The first principal component accounted for 94.7% of the variation and the second component explained only 2.2% of the variation (**Figure 2**). In addition, it was observed that features clustering close to zero had low variance among the populations, whilst the features that were distributed throughout the plot presented the highest variance. Based on this analysis, only the six most distant features from zero on the X axis were chosen (A8, B17, G16, I9, J8, O2) since the first component explains most of the variation.

Furthermore, the magnitude of the variance for the full set of features was examined across the 27 genotypes. Three species-specific features were identified (B15, C2, M2) that were not previously detected by PCA since they had low means across the fingerprints. These results imply that PCA was only able to detect the features with high variance and high mean, excluding polymorphic sequences in the dataset with high variances and low mean signal intensity among the fingerprinted samples. The three features identified presented low signal strength for all *E. purpurea* lines analyzed, thus it differentiated *E. purpurea* from the other fingerprinted species.

### Correlation of the Genetic Profile with Metabolic Profiling

The signal strength of each of the 283 features was correlated with each of the 43 lipophilic metabolites identified in the roots of the 19 genotypes shared between the present study and the previous metabolomic profiling [21]. Positive correlation was found between the signal strength of feature H9, L2 and M8 and the relative content of 2,4 diene alkamides and Chen alkamide. In addition, the signal strength of I18 and F15 had significant positive correlations with the relative content of monoene alkamides. Signal of F15 was also found to correlate with ketone 24 **(** **Table 2** **)**.

**Table 2:** Significant correlations among the signal of each of the 283 features and the relative abundance of 43 lipophilic metabolites.

Compound	H9	L2	M8	I18	F15
**Amide 1** a		0.82**			
Sig. (2-tailed)		0.00			
**Amide 2** a	0.65**	0.81**			
Sig. (2-tailed)	0.00	0.00			
**Amide 3** a	0.87**		0.74**		
Sig. (2-tailed)	0.00		0.00		
**Amide 5**				0.54*	
Sig. (2-tailed)				0.02	
**Amide 7** a	0.86**	0.79**	0.75**		
Sig. (2-tailed)	0.00	0.00	0.00		
**Amide 9**				0.57*	
Sig. (2-tailed)				0.02	
**Amide 10** a				0.59**	
Sig. (2-tailed)				0.00	
**Amide 11** a				0.48*	
Sig. (2-tailed)				0.04	
**Amide 12** b				0.49*	
Sig. (2-tailed)				0.03	
**Amide 13** b				0.59**	
Sig. (2-tailed)				0.00	
**Amide 14** b				0.71**	
Sig. (2-tailed)				0.00	
**Amide 15**					0.79**
Sig. (2-tailed)					0.00
**Amide 16** b				0.75**	
Sig. (2-tailed)				0.00	
**Amide 17** b				0.59**	
Sig. (2-tailed)				0.00	
Chen alkamide	0.92**	0.71**	0.70**		
Sig. (2-tailed)	0.00	0.00	0.00		
**Ketone 22**			−0.56*		
Sig. (2-tailed)			0.02		
**Ketone 24**			−0.61**		0.56*
Sig. (2-tailed)			0.00		0.02

a 2,4-diene alkamides.

b Monoene alkamides.

** Correlation is significant at the 0.01 level.

* Correlation is significant at the 0.05 level.

**Figure 3:**
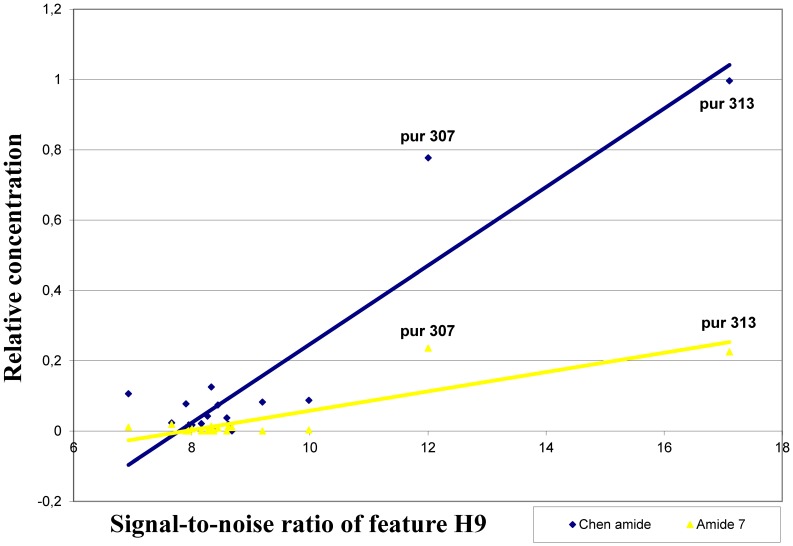
Correlation among the signal strength of feature H9 and the relative content of alkamides. Significant positive correlations are shown for signal strength of feature H9 and the content of chen alkamide and amide 7.

**Figure 4:**
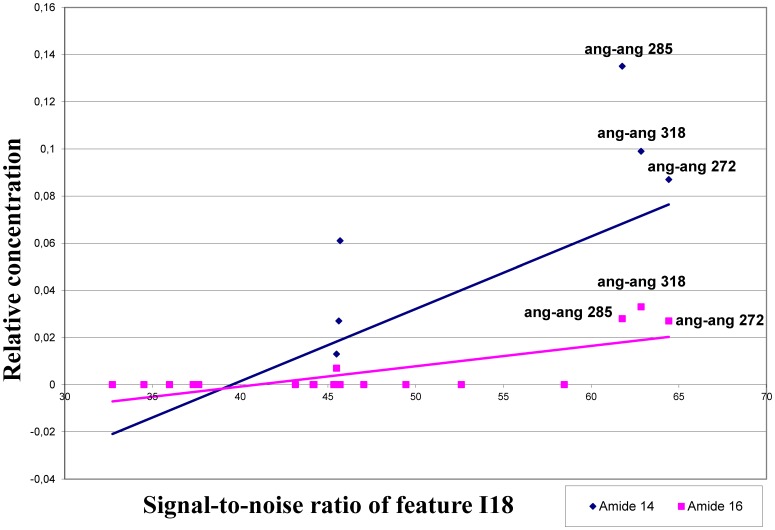
Correlation among the signal strength of feature I18 and the relative content of monoene alkamides. Significant positive correlations are shown for signal strength of feature H9 and the content of amide 14 and 16.

The most significant correlations were found between the signal strength of feature H9 and the content of chen alkamide (r = 0.92; P<0.01), amide 3 (r = 0.87; P<0.01) and amide 7 (r = 0.87; P<0.01). The signal strength of H9 and the content of the amides were the highest for PI631307 and PI631313 lines **(** **Figure 3** **)**, which are the only two *E. purpurea* lines used in both studies. Similar positive correlations were also found between the signal strength of H9, L2 and M8 and the content of amide 2, 3, 7 and chen alkamide. Furthermore, the signal strength of I18 and content of the amides 14 and 16 were the highest for the *E. angustifolia* var. *angustifolia* genotypes **(** **Figure 4** **)**. This indicates that the signal strength of features H9, L2 and M8 have a similar pattern of variation as the relative content of the amides 2, 3, 7 and chen amide in the two *E. purpurea* genotypes analyzed. Similarly, the signal strength of the feature I18 has a similar pattern of variation as the content of amide 14 and 16 in the *E. angustifolia* var. *angustifolia* genotypes analyzed.

### The Sequence Identity of the most Interesting Features

The amplification products of the six features chosen by PCA and the three species-specific features were sequenced along with the amplification products of the five features whose signal strength was found to be positively correlated to the content of lipophilic metabolites. Five features (C2, G16, I9, J8 and M2) had significant alignments with putative retrotransposon sequences **(** **Table 3** **)**. For the feature I9 [EMBL: HF585707], only 14% of the sequence was found to be 78% identical to a retrotransposon *RIRE1*. While for feature M2 [EMBL: HF585711], 51% of the sequence was found to be 78% identical to a Ty3/*gypsy*-like retrotransposon. Features C2 [EMBL: HF585703], G16 [EMBL: HF585705] and J8 [EMBL: HF585709] significantly matched to the same retrotransposon locus. However, only G16 and J8 sequences partially overlapped by 95 bp as it was found after performing sequence alignment (blastN) of the three sequences. In addition, both of these features were found to have different patterns of variation since no significant correlation was found (r = −0.004, P>0.05) after using correlation bivariate.

**Table 3:** Predicted locus/function of the 14 sequenced SDA features using blastN program through National Centre of Biotechnology Information (www.ncbi.nlm.nih.gov).

Feature ID	Length (bp)	Matching database entry	Putative identity	E Value
A8^a^	323		No hits	NA
B15^b^	252		No hits	NA
B17^a^	344	EL419699.1	*Helianthus ciliaris* uncharacterized cDNA sequence	2e-11
C2^b^	341	FJ791047.1	*Helianthus annuus* retrotransposon HA7, complete sequence	2e-18
F15^c^	744	EU362851.1	Ambrosia asymptomatic virus 2 UKM-2007 isolate05TGP00321.Bad4 ORF1–2 gene, partial cds	6e-54
G16^a^	328	FJ791047.1	*Helianthus annuus* retrotransposon HA7, complete sequence	4e-21
H9^c^	249		No hits	NA
I9^a^	550	D85597.1	*Oryza australiensis* retrotransposon RIRE1 DNA	7e-08
I18^c^	447		No hits	NA
J8^a^	300	FJ791047.1	*Helianthus annuus* retrotransposon HA7, complete sequence	2e-11
L2^c^	643	JN021935.1	*Helianthus annuus* cutivar HA383 clone BAC 0516M24, complete sequence	4e-43
M2^b^	829	GQ367282.1	*Helianthus petiolaris* isolate 94XPET9 retrotransposonTy3/gypsy-like reverse transcriptase-like gene,partial sequence	1e-95
M8^c^	454		No hits	NA
O2^a^	360		No hits	NA

The best match is shown as the putative identity for each sequence. E-value was regarded as significant if <1e-10. NA indicates the absence of significant data.

a Features that were chosen by PCA.

b Features that were found to have low signal strength for *E. purpurea*.

c Features whose signal strength correlated significantly with the content of lipophilic metabolites.

Additionally, two other features could also be related to retrotransposon sequences. Feature L2 [EMBL: HF585710] matched to the sequence of a bacterial artificial chromosome (BAC) clone. Interestingly, 74% of this feature sequence was found to be 76% identical to the sequence of a BAC clone that was found between a *copia*- and a *gypsy*-like retrotransposon. However this fragment did not have its own identity. While for feature F15 [EMBL: HF585704], 74% of the sequence was found to be 70% identical to an open reading frame (ORF) 1–2 gene of an Ambrosia asymptomatic virus. Further, feature B17 [EMBL: HF585702] corresponded to an uncharacterized cDNA sequence and the other six remaining features were not recognized as known DNA sequences or proteins [EMBL: HF585700, HF585701, HF585706, HF585708, HF585712, HF585713] **(** **Table 3** **)**.

It is important to take into account that among these 14 features there was more than one feature that showed specificity to the same species, implying that among these 14 features there are some of them that have the same patterns of variation across the 27 genotypes. Pearson bivariate correlation performed among the 14 features **(data not shown)**, indicated that there were positive significant correlations between I9 and A8 (r = 0.85, P<0.01), between I9 and O2 (r = 0.83, P<0.01), and between M8 and H9 (r = 0.7, P<0.01). It is important to note that although these features were highly correlated may not necessarily imply that they possess high sequence similarity. However, A8, H9 and O2 were eliminated from the set of polymorphic features since I9 and M8 could explain most of the variation found in them.

**Figure 5:**
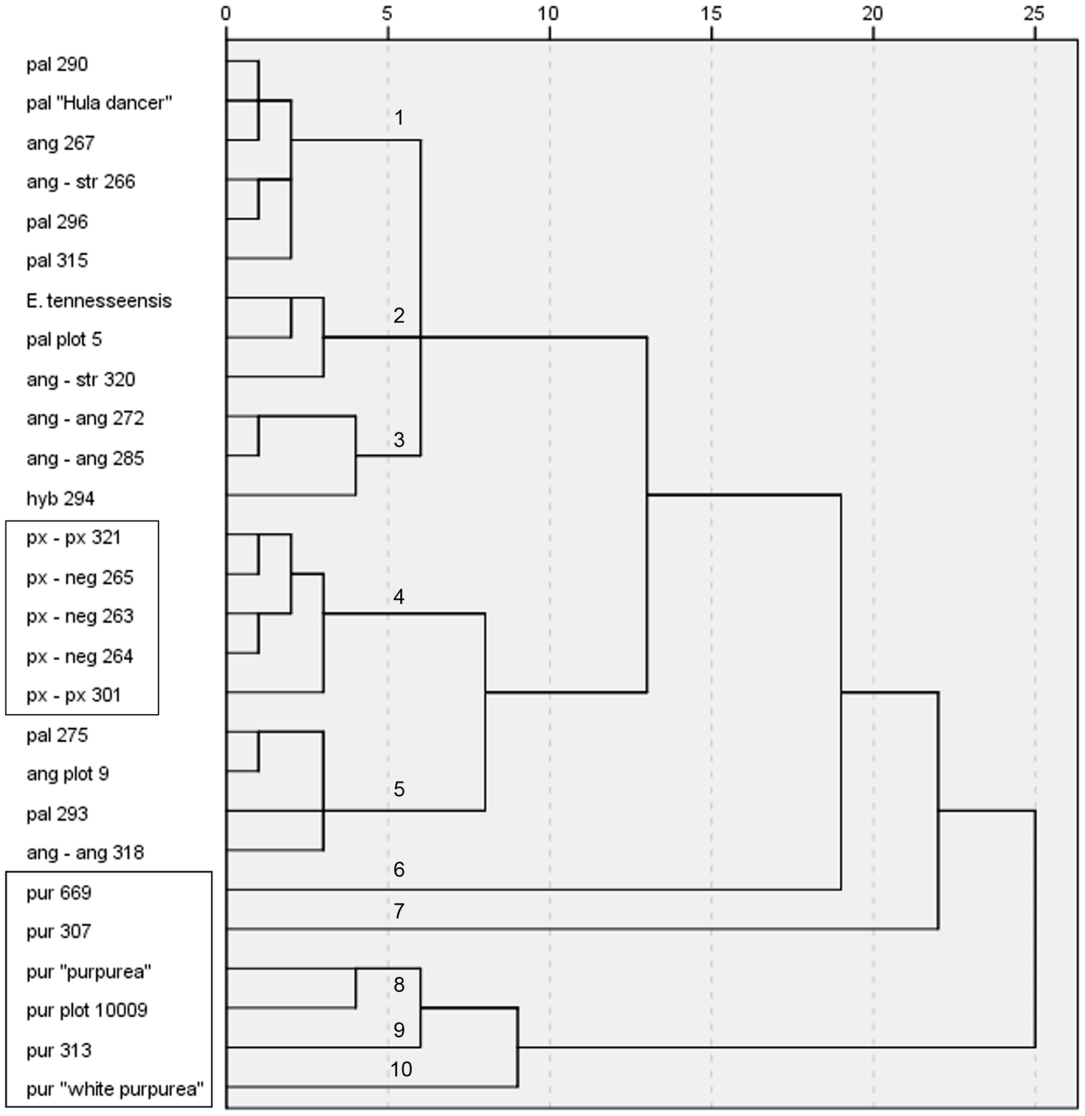
Dissimilarity dendrogram for the 27 genotypes using only the eleven most discriminatory features. The steps of the dendrogram (Squared Euclidian distance, between groups linkage) show the combined clusters and the values of the distance coefficients at each step; the values have been rescaled to numbers between 0 and 25, preserving the ratio of the distances between the steps. The equivalents of the abbreviated names used for each of the genotypes are shown in Table 1.

Based on the above analysis, only 11 features were selected to perform a second hierarchical cluster **(** **Figure 5** **)**. A comparison of this new dendrogram with the original one constructed with the full set of features **(** **Figure 1** **)** indicated that the clustering of the 27 genotypes was consistent with the major clusters obtained with the full data set. For instance, the *E. purpurea* genotypes could be clearly differentiated from the other species in the new dendrogram as found in the original. Similarly, all *E. paradoxa* genotypes were found in a single cluster (Cluster 4) and in addition four of the seven *E. pallida* genotypes were found in Cluster 1. Consequently, it may be inferred that these 11 features are the most discriminatory features for fingerprinting of these 27 genotypes.

## Discussion

*Echinacea* are among the top 10 selling herbal medicines in the U.S. and Europe. In the U.S. alone, an annual sales of *Echinacea* products has been estimated to be from more than $200 to $300 million [2], [4]. However, this increase in the market has revealed problems in quality control. For instance, species misidentification remains problematic since there are many morphological similarities between species. We report the construction of an *Echinacea-*specific SDA capable to fingerprint closely related species and accessions of this genus. Furthermore, we discuss the usefulness of this SDA to assess genetic relationships among *Echinacea* species and highlight its ability to identify potential nuclear molecular markers that could assist in future phylogenetic analyses.

### Subtraction Efficiency

The subtraction technique was able to eliminate about 97% of common DNA sequences between the tester and the driver pool. This subtraction efficiency was higher than the 88% efficiency obtained for the *Salvia*-specific SDA [20] and identical to the one obtained for the prototype SDA for angiosperms [16], where 12 (3%) features were found to be positively hybridized to the driver DNA.

The subtraction efficiency of the *Echinacea*-specific SDA was higher than that obtained for the *Salvia*-specific SDA, possibly due the increase in the tester:driver ratio from 1∶30 to 1∶60. The (1∶30) ratio used in the *Salvia* study was also used for the angiosperm SDA subtraction [16] which effectively eliminated the common sequences between the tester (angiosperm) and driver (non-angiosperm). However, the *Echinacea* and *Salvia* subtractions were in a sense more stringent compared to the subtraction between angiosperm and non-angiosperm as these subtractions were performed at the genera level. Therefore, the sixty-fold excess of driver added during the *Echinacea* subtraction may have removed most of the sequences that were homologous between the *Echinacea* and driver pools resulting in 97% subtraction efficiency. It has been suggested earlier that a higher concentration of driver DNA will theoretically subtract the sequences that are partially homologous between the tester and driver, thus enriching for those highly specific sequences [26]. Comparatively, the thirty-fold excess of driver used in the *Salvia* study may have not been sufficient to eliminate such sequences resulting in only 88% subtraction efficiency. Consequently, future subtractions made at the genera level should be performed using a 1∶60 tester:driver ratio.

### Capacity of *Echinacea*-specific SDA to Fingerprint and Assess the Genetic Relationships among Twenty-seven *Echinacea* Lines

The two hierarchical clusters performed demonstrated the ability of the SDA to fingerprint closely related species and accessions (genotypes) within the species. For instance, it was possible to clearly differentiate *E. purpurea* and *E. paradoxa* from the other three species fingerprinted (**Figure 1** **and** **5**).

Additionally, the *Echinacea*-SDA was capable of fingerprinting genotypes that were not used in its construction. For instance, it was possible to fingerprint *E. angustifolia* (ang plot 9), *E. pallida* (pal plot 5 and pur plot 10009) and a hybrid of *E. paradoxa* var. *paradoxa* with *E. pallida* (hyb 294), even though they were not part of the original subtraction pool. For example, the *E. purpurea* plot 10009 genotype grouped with the other genotypes from the same species in both dendrograms (**Figure 1** **and** **5**). This indicates the broad applicability of this genera-specific SDA to fingerprint even those *Echinacea* genotypes that were not used in the construction process.

Further, the hierarchical analyses generated (species were labeled on the basis of McGregor’s classification) provided better support to Binns’s classification for three primary reasons.

Firstly, *E. purpurea* was clearly differentiated from the other four species in the hierarchical cluster analyses performed with full set of features (**Figure 1**) and the one performed with the 11 most useful features (**Figure 5**). **Figure 1** shows a distance threshold of more than 20 between *E. purpurea* and the cluster that contains the other three species. This result agrees with the conclusions from Binns et al. [7] study where two major divergent taxa within *Echinacea* were found. In this classification, *E. purpurea* is the only member in the subgenus *Echinacea* and the subgenus *Pallida* included all other taxa.

Secondly, there was no clear differentiation between the *E. pallida* and *E. angustifolia* genotypes. As shown in **Figure 1** **and** **5**, the seven genotypes from each of these two species did not cluster as expected according to the species or varieties from which they belonged [8]. For instance in **Figure 1**, the *E. pallida* (PI631293 and PI631275) and *E. angustifolia* (PI 631318 and Plot 9) genotypes were found in cluster 2 which was clearly differentiated from cluster 3 and 4 that contained the other genotypes of these two species. Again, this result is more in agreement with Binns et al. [7] classification, where *E. angustifolia* is a variety of *E. pallida* which contains five varieties [*E. pallida* (Nutt.) Nutt. var. *angustifolia* (DC.) Cronq, *E. pallida* (Nutt.) Nutt. var. *pallida*, *E. pallida* (Nutt.) Nutt. var. *sanguinea* (Nutt.) Gandhi & R.D. Thomas, *E. pallida* (Nutt.) Nutt. var. *simulata* (McGregor) Binns, B.R. Baum, & Arnason and *E. pallida* var. *tennesseensis* (Beadle) Binns B.R.Baum, & Arnason].

Thirdly, the current results could not support the classification of *Echinacea* into varieties as suggested by McGregor [8]. McGregor recognized four varieties [*E. angustifolia* DC. **var.** ***angustifolia***, *E. angustifolia* DC. **var.** ***strigosa*** McGregor, *E. paradoxa* (Norton) Britton **var.** ***neglecta*** McGregor and *E. paradoxa* (Norton) Britton **var.** ***paradoxa***] of which only the genotypes belonging to *E. paradoxa* var. *neglecta* clustered together **(** **Figure 1** **)**. Comparatively, Binns recognized eight varieties [five of *E. pallida*, *E. atrorubens* (Nutt.) Nutt. **var.** ***atrorubens***, *E. atrorubens* (Nutt.) Nutt. **var.** ***paradoxa*** (J.B. Norton) Cronq. and *E. atrorubens* (Nutt.) Nutt. **var.** ***neglecta*** (McGregor) Binns, B.R. Baum, & Arnason]. However it was not possible to support this classification entirely since some of the taxa could not be included in this study (*E. atrorubens*, *E. laevigata*, *E. sanguinea* and E. *simulata*) due to quarantine restrictions.

Although most of the results explained above support the classification by Binns et al. [7], the SDA profile could not unequivocally support the division of *Echinacea* into four species or eight varieties due to the number of species used. Therefore, further studies that include all species are needed in order to elucidate the genetic relationships for all *Echinacea* species. To date, it has not been possible to reconstruct the genetic and evolutionary relationships of this genus. The main limitations are the population sampling and the use of techniques such as AFLP and Random Amplified Polymorphic DNA (RAPD) that make the assumption that co-migrating fragments are homologous, thus limiting its applications for phylogenetic analyses [10], [11], [27]. In addition, the use of chloroplast and nuclear loci, which are commonly used for phylogenetic studies, were unable to resolve the species level relationships due to the low levels of molecular divergence found in these loci [12]. SDA offers a good alternative for DNA-based phylogenetic analysis; however the results from this study provide an incomplete assessment of the phylogenetic relationship of the genus since not all the species were analyzed.

### Correlations between the Genetic and Chemical Profiles

The significant positive correlations found between the hybridization profiles of H9, L2 and M8 and the content of 2,4 diene alkamides in the accessions analyzed may be attributed to the fact that 2,4 dienoic acid unit is present in higher amounts in *E. purpurea* [6] and the signal strength of these features was relatively higher for *E. purpurea* genotypes. Therefore, these three features could serve as good markers for *E. purpurea*. However they may not be considered as potential markers for 2,4 diene alkamides since the signal strength and the relative abundance of the amides do not share a similar pattern of variation for all other species. The same problem was found for feature I18, where the signal strength of I18 has a similar pattern of variation as the relative content of amides 14 and 15 only for *E. angustifolia* var. *angustifolia* and not with the other species. Consequently, the significant correlations found could indicate that these loci may potentially be species-specific markers rather than markers linked to genes responsible for the production of these bioactive compounds.

It is important to note that even though this study used the same accessions as the metabolic profiling study [21], and sourced these accessions from the same germplasm collection, different plants were used for each study. Previous studies have found that populations and cultivars of *Echinacea* are genetically heterogeneous [27], [28]. Therefore, the fact that these two studies were performed on different plants may be a possible reason for the different patterns of variation among signal strength of the features and the relative content of the lipophilic metabolites. Previous studies have found that DNA molecular markers are useful for predicting the phytochemical concentration of *Echinacea* plants. AFLP DNA fingerprints were found to be statistically significant predictors of cichoric acid and dodeca-2E, 4E, 8Z, 10E/Z-tetraenoic acid isobutyl amide (amide 8 and 9) in cultivated *E. purpurea* and some related wild species [29]. In addition, RAPD markers were able to predict polyphenol content in aerial parts of *E. purpurea* [30]. However, to date, no study has performed a correlation analysis that includes all *Echinacea* species. Future studies could perform parallel chemical and molecular profiles with all the species in order to find if species-specific markers could also be associated to the production of bioactive compounds, since the abundance of the compounds varies greatly depending on the species [21].

### Sequence Identity of the most Interesting Features

Out of fourteen features sequenced, five corresponded to known retrotransposon loci and two others may be also related to retrotransposon sequences. Retrotransposons are mobile genetic elements which can be classified in two clearly separate groups, the long terminal repeat (LTR) retrotransposons and non-LTR retrotransposons [31]. Features C2, G16 and J8 matched to the same database entry, *Helianthus annuus* retrotransposon HA7 (FJ791047.1) which is a putative LTR [32]. Feature I9 and M2 also matched to LTR retrotransposons. Feature I9 had a good match to a retrotransposon named *RIRE1* (for Rice Retroelement; D85597.1) [33], while M2 significantly matched to a *Ty3/gypsy*-like LTR retrotransposon (GQ367282.1) [34]. LTR- retrotransposons have been found to be more prevalent in plant genomes (can comprise about 50% of the nuclear DNA) and have been found to play a major role in the expansion of the genome size [35]. For instance, it has been found that *RIRE1* caused an increase in size of about 11 Mb in *Oryza australiensis* [33]. The high abundance of LTR retrotransposons in the genome, their ubiquitous nature and their activity in creating genomic diversity by stably integrating large DNA segments into dispersed chromosomal loci, make this group of retrotransposons ideal for development as molecular markers [31]. Previous studies have found that it is possible to use retrotransposons for fingerprinting cultivated rice species [36], to obtain genomic diversity patterns of *Pisum* [37] and to elucidate the evolutionary events of three *Helianthus* hybrid species independently derived from two parental species [34]. The results obtained in the present study suggest that LTR retrotransposons are highly polymorphic in *Echinacea*; therefore the five loci that matched to known retrotransposons have the potential to become retrotransposon-based molecular markers useful for fingerprinting and studying diversity patterns in *Echinacea*.

Furthermore, the feature F15 significantly matched to an ORF 1–2 gene of an Ambrosia asymptomatic virus (EU362851.1). This virus specifically was identified as a badnavirus which belongs to the Caulimoviridae family [38]. Caulimoviridae are known to be plant pararetroviruses that replicate their genome through a process of reverse transcription [39]. There have been previous reports on plant viruses involving integration into the plant genome as part of their infection cycle [40]. Therefore, this could be a case of an ‘endogenous’ viral retroelement where after integration into the genome they evolve in a manner of a pseudogene accumulating inactivating mutations [41]. If the distribution of this pseudogene varies among the species of *Echinacea*, as the SDA results suggest, then the integration event could shed some light on how the different species diverged.

To the best of authors’ knowledge, this is the first time retrotransposon sequences have been used to fingerprint *Echinacea*. Five out of the eleven most discriminatory features matched to known retrotransposons which were found to discriminate among species and accessions of *Echinacea* (**Figure 5**). Moreover, if further analyses confirm that feature F15 is an endogenous virus whose sequence is polymorphic among the *Echinacea* species, then this feature could also become a potential molecular marker. Therefore, these six retroelements together with L2 feature, which matched to a sequence found between a *copia*- and a *gypsy*-like retrotransposons, could be employed to elucidate the relationships among the *Echinacea* species. For example, in *Helianthus annuus* L., it has recently been found that a vast majority of LTR retrotransposon insertions have likely occurred since the origin of this species; inferring that retrotransposons have played an important role in the evolution of this species [42]. *Helianthus* is in the same family (Asteraceae) as *Echinacea*; therefore it is likely that retrotransposon insertions have also contributed to *Echinacea* genome evolution. However, instead of using the entire array, specific primers could be developed for amplification of selected sequences within species. Then, isolation and sequence of these products could reveal which microstructural changes (insertion, deletion, inversion) are responsible for the segregation of these SDA features. Therefore, these sequences may lead to the development of nuclear molecular markers for sequence-based analyses which may provide a good alternative for a nuclear DNA-based phylogenetic analysis.

In summary, the efficient enrichment of specific sequences during subtraction (97%) made it possible to obtain a set of unique sequences for *Echinacea*. The *Echinacea*-SDA clearly differentiated *E. purpurea* from the other species; however no clear differentiation was observed between the *E. pallida* and *E. angustifolia* genotypes. Therefore, these results provided better support to the classification proposed by Binns et al. [7]. However, due to the limited number of species used in this study, it was not possible to unequivocally support the division of *Echinacea* into four species and eight varieties as proposed by this morphometric classification. Moreover, five retrotransposon sequences were identified to be polymorphic among the 27 genotypes, together with a possible endogenous pararetrovirus, which can be explored for phylogenetic analyses in the future.

## Supporting Information

Table S1 **Description of the angiosperm and non-angiosperm species used for DNA extraction and development of genome representations for preparing the** ***Echinacea*** **-specific SDA.**
(DOCX)
