# Supplementary material for: Subtracted Diversity Array Identifies Novel Molecular Markers Including Retrotransposons for Fingerprinting Echinacea Species
Source: PLoS One. 2013 Aug 5;8(8):e70347. doi: 10.1371/journal.pone.0070347 (PMC3734018; doi:10.1371/journal.pone.0070347)
Supplement: Table S1 — Description of the angiosperm and non-angiosperm species used for DNA extraction and development of genome representations for preparing the Echinacea -specific SDA. (DOCX) [file pone.0070347.s001.docx]

**Table S1.** Description of the angiosperm and non-angiosperm species used for DNA extraction and development of genome representations for preparing the *Echinacea*-specific SDA.

| **REPRESENTATIONS** |  | **SPECIES** |  |
| --- | --- | --- | --- |
| **NON ANGIOSPERMS**  **(25 species)** | *Adiantum raddianum*  *Azolla* sp.  *Blechnum chambersii*  *Blechnum fluviatile*  *Bryum billardieri*  *Catagonium nitens*  *Cyathea cooperi*  *Cyathophorum* sp.  *Dawsonia superba* | *Dicksonia antarctica*  *Equisetum hyemale*  *Ginkgo biloba*  *Grammitis billardieri*  *Hymenophyton flabellatum*  *Marchantia* sp.  *Microsorum pustulatum*  *Polystichum proliferum*  *Racopilum cuspidigerum v*ar.c*onvolutaceum* | *Riccardia eriocaula*  *Selaginella* sp.  *Sphagnum australe*  *Sticherus tener*  *Thuidium* sp.  *Weymouthia* *cochlearifolia*  *Wollemia nobilis* |
| **MAGNOLIIDS**  **(6 species)** | *Cinnamomum verum*  *Houttuynia cordata* | *Illicium anisatum*  *Magnolia denudate* | *Nymphaea gigantea*  *Peumus boldus* |
| **MONOCOTS**  **(23 species)** | *Acorus calamus*  *Acorus gramineus*  *Aloe vera*  *Bambusa beecheyana*  *Bletilla striata*  *Coix lacryma-jobi*  *Colocasia esculenta*  *Curcuma longa* | *Dioscorea polystacha*  *Fritillaria thunbergii*  *Iris domestica* (syn. *Belamcanda chinensis*)  *Iris versicolor*  *Lilium longiflorum*  *Lomandra longifolia*  *Ophiopogon japonicus*  *Pinellia cordata* | *Polygonatum multiflorum*  *Ruscus aculeatus*  *Serenoa repens*  *Trachycarpus fortunei*  *Zea mays*  *Zephyranthes sp.*  *Zingiber officinale* |
| **EUDICOTS NOT PLACED IN EITHER THE ROSIDS OR**  **ASTERIDS SUBCLADES**  **(19 species)** | *Aconitum carmichaelii*  *Aquilegia* sp.  *Berberis fortunei* (syn. *Mahonia fortunei*)  *Berberis japonica* (syn. *Mahonia japonica*)  *Buxus sempervirens* | *Chelidonium majus*  *Clematis hexapetala*  *Clematis montana*  *Clematis serratifolia*  *Clematis songarica*  *Dianthus caryophyllus*  *Dianthus superbus* | *Eschscholzia californica*  *Grevillea robusta*  *Gypsophila oldhamiana*  *Hamamelis virginiana*  *Phytolacca acinosa*  *Ranunculus* sp.  *Rumex crispus* |
| **ROSIDS**  **(33 species)** | *Abutilon theophrasti*  *Agrimonia eupatoria*  *Agrimonia pilosa*  *Albizia julibrissin*  *Alchemilla xanthochlora*  *Althaea officinalis*  *Armoracia rusticana*  *Astragalus membranaceus*  *Baptisia tinctoria*  *Allocasuarina* sp.  *Catha edulis* | *Citrus aurantium*  *Citrus reticulata*  *Crataegus monogyna*  *Dichroa febrifuga*  *Filipendula ulmaria*  *Firmiana simplex*  *Glycyrrhiza glabra*  *Glycyrrhiza uralensis*  *Gynostemma pentaphyllum*  *Humulus lupulus*  *Hypericum perforatum* | *Isatis tinctoria*  *Oenothera biennis*  *Oenothera odorata*  *Oxalis pes-caprae*  *Passiflora edulis*  *Pelargonium sp.*  *Poncirus trifoliata*  *Rosa rugosa*  *Ruta graveolens*  *Sophora flavescens*  *Urtica dioica* |
| **ASTERIDS**  **(37 species)**  **Excluding Asteraceae** | *Angelica archangelica*  *Angelica dahurica*  *Bacopa monnieri*  *Camellia sinensis*  *Centella asiatica*  *Coffea arabica*  *Digitalis purpurea*  *Forsythia suspensa*  *Glechoma hederacea*  *Hedeoma pulegioides*  *Hyssopus officinalis*  *Ilex paraguariensis*  *Impatiens* sp. | *Leonourus sibiricus*  *Leonurus cardiaca*  *Lycium barbarum*  *Melissa officinalis*  *Mentha × piperita*  *Mentha arvensis var. piperascens*  *Malinv. ex L. H. Bailey*  *Mentha pulegium*  *Mentha spicata*  *Nepeta cataria*  *Perilla frutescens*  *Plantago major*  *Platycodon grandiflorus* ‘Apoyama’ | *Prunella vulgaris*  *Sambucus nigra*  *Scrophularia nodosa*  *Scutellaria lateriflora*  *Stachys officinalis*  *Symphytum* *officinale*  *Thymus vulgaris*  *Valeriana officinalis*  *Verbascum thapsus*  *Vitex agnus-castus*  *Withania somnifera* |
